# The relationship between numerical magnitude processing and math anxiety, and their joint effect on adult math performance, varied by indicators of numerical tasks

**DOI:** 10.1007/s10339-024-01186-0

**Published:** 2024-04-22

**Authors:** Monika Szczygieł, Mehmet Hayri Sarı

**Affiliations:** 1 Institute of Psychology, Jagiellonian University in Kraków, Kraków, Poland; 2 Faculty of Education, Nevşehir Hacı Bektaş Veli University, Nevşehir, Türkiye

**Keywords:** Symbolic magnitude processing, Non-symbolic magnitude processing, Mental number line, Math anxiety, Math performance

## Abstract

According to the hypothesis of Maloney et al. (Cognition 114(2):293–297, 2010. https://doi.org/10.1016/j.cognition.2009.09.013), math anxiety is related to deficits in numerical magnitude processing, which in turn compromises the development of advanced math skills. Because previous studies on this topic are contradictory, which may be due to methodological differences in the measurement of numerical magnitude processing, we tested Maloney et al.’s hypothesis using different tasks and their indicators: numerical magnitude processing (symbolic and non-symbolic comparison tasks: accuracy, reaction time, numerical ratio, distance and size effects, and Weber fraction; number line estimation task: estimation error), math anxiety (combined scores of learning, testing, math problem solving, and general math anxiety), and math performance. The results of our study conducted on 119 young adults mostly support the hypothesis proposed by Maloney et al. that deficiency in symbolic magnitude processing is related to math anxiety, but the relationship between non-symbolic processes and math anxiety was opposite to the assumptions. Moreover, the results indicate that estimation processes (but not comparison processes) and math anxiety are related to math performance in adults. Finally, high math anxiety moderated the relationship between reaction time in the symbolic comparison task, reaction time in the non-symbolic comparison task, numerical ratio effect in the symbolic comparison task, and math performance. Because the results of the joint effect of numerical magnitude processing and math anxiety on math performance were inconsistent, this part of the hypothesis is called into question.

## Introduction

Symbolic and non-symbolic magnitude processing and math anxiety are considered to be important predictors of math performance (Braham and Libertus 2018; Ramirez et al. 2016). However, relatively little is known about the relationship between numerical magnitude processing and math anxiety and their combined effect on math performance. Recently, Maloney et al. (2010) formulated the hypothesis that math anxiety develops because of deficits in numerical magnitude processing, which in turn, compromises the development of advanced mathematical skills. It should be noted that this hypothesis is difficult to verify in one cross-sectional study. Testing whether math anxiety stems from poor numerical processing and in turn affects math performance requires longitudinal studies, preferably from early childhood to adulthood, and control for multiple covariates. However, Maloney et al. (2010) hypothesis is commonly verified by examining the relationship between numerical magnitude processing and math anxiety, by comparison of the level of numerical magnitude processing in low and high math anxiety individuals, and by testing the mediation or moderation effect of math anxiety in the relationship between numerical magnitude processing and math performance. Research on this topic is most often conducted in a cross-sectional design, mainly in adults, and previous research results on this topic are contradictory.

Conflicting research results may stem from different definitions of numerical magnitude processing and the use of different methodologies for their measurement. Indeed, there is an ongoing debate in the field of mathematical cognition about the uniformity of numerical systems, the cognitive processes involved in processing numerical quantities, and the validity of magnitude processing indicators (Dietrich et al. 2015; Krajcsi 2017; Krajcsi & Szűcs 2022; Krajcsi et al. 2023; Lyons et al. 2012, 2015; Piazza et al. 2004; Price et al. 2012; Smets et al. 2014). Therefore, we are interested in verifying the hypothesis regarding the relationship between numerical magnitude processing and math anxiety, and their joint effect on math performance, taking into account various numerical magnitude processes (symbolic vs. non-symbolic; comparison vs. estimation) and ways of measuring them (comparison tasks and its indicators: accuracy, reaction time, numerical ratio effect, distance effect, size effect, and Weber fraction; number line estimation task and its indicator: estimation error). Below, we present the theoretical foundations of numerical magnitude processing, math anxiety, and all relevant research on the relationships between them and their relationships to math performance. We present methodological details of previous relevant studies to highlight possible differences in results due to study design.

## Numerical magnitude processing

Although there are many concepts of numerical magnitude processing, generally it is understood as the mental manipulation of quantitative information of either non-symbolic quantities (e.g., dot arrays) or symbolic numbers (e.g., Arabic digits; Chen et al. 2021; Schneider et al. 2017, 2018a, 2018b). Numerical magnitudes have spatial representations, which are described via the metaphor of the mental number line (Dehaene 2001, 2011; Restle 1970; Schneider et al. 2018a), in which numbers are represented on a left-to-right continuum (Dehaene et al. 1993; Zorzi et al. 2002). Representing and processing of magnitudes is supported by an approximate number system (ANS; Cantlon et al. 2009; Dehaene 2001; 2011). ANS is a language-independent and innate system that is shared across many species (Pica et al. 2004; Wynn 1992) and represents quantities approximately (Feigenson et al. 2004; Li et al. 2018). The precision of the non-symbolic numerical system increases with age (Halberda et al. 2008). Accordingly, the symbolic number representation system is an acquired and language-dependent system that represents quantities precisely (Dehaene 2001; Li et al. 2018) and develops gradually over the school years and allows for the processing of discrete numbers (Marinova & Reynvoet 2020; Schleepen et al. 2016). There is an ongoing debate about the relationship between the symbolic and non-symbolic systems (Krajcsi, et al., 2022; Price et al. 2012; Smets et al. 2014) as both are viewed as either related (Piazza et al. 2004) or independent (Dietrich et al. 2015; Honoré & Noël, 2016; Lyons et al. 2012, 2015). Moreover, previous research results provide arguments that comparison and estimation processes are unrelated (Guillaume et al. 2016; Sasanguie & Reynvoet 2013) or weakly related (Tokita & Hirota 2021). Therefore, the search for correlates of numerical processing of quantities requires taking this diversity into account.

Non-symbolic and symbolic numerical abilities are most often measured with number line estimation and magnitude comparison tasks (Schneider et al. 2018b). The number line estimation task requires participants to indicate the position of a given number on a line that is anchored at both ends (e.g., from 0 to 100; Núñez-Peña et al. 2019; Pantoja et al. 2020). The sum of errors (the difference between the target and marked point on a number line in each trial) is the most used indicator of the number line estimation task. To decide which of two quantities is larger, the comparison task relies on the comparison of Arabic numbers or dot arrays, presented in paired, sequential, or intermixed ways (Price et al. 2012). Accuracy (the sum of correct answers; Landerl et al. 2004), reaction time (the average reaction time for correct answers; Schwenk et al. 2017), individual Weber fraction (*W* is the internal Weber fraction, which determines the degree of accuracy of the representation of the internal quantity; Krajcsi 2020; Pica et al. 2004), numerical ratio effect (it is easier to process number pairs with higher ratios than pairs with smaller ratios; Price et al. 2012), numerical distance effect (it is easier to compare number pairs that are further apart; Maloney et al. 2010), and numerical size effect (for a constant distance, it is easier to compare smaller number pairs; Hohol et al. 2020) are the most commonly used indicators of the performance of comparison tasks. Although these indicators of comparison tasks are considered to reflect the precision of numerical representations, they are usually more or less correlated to each other (e.g., numerical ratio, distance, and size effects are related to each other; Krajcsi 2020; Weber fraction and numerical ratio effect are not; Price et al. 2012).

Despite the diversity of basic numerical systems (symbolic vs. non-symbolic) and task related processes (estimation vs. comparison), the results of many previous studies suggest a reliable relationship between numerical magnitude processing and math performance in children, adolescents, and adults (Schneider et al. 2017; 2018a, 2018b). However, the relationship between math performance and number line estimation is stronger than the relationship between magnitude comparison and math performance (Schneider et al. 2018b); also, the strength of the correlation with math performance is significantly higher for the symbolic comparison task than for the non-symbolic. Additionally, the associations between math performance and magnitude comparison tasks decrease very slightly with age (Schneider et al. 2017), whereas the relationship between math performance and number line estimation increases slightly with age (Schneider et al. 2018a).

## Math Anxiety

Math anxiety can be defined as “[…] a feeling of tension and anxiety that interferes with the manipulation of numbers and the solving of mathematical problems in a wide variety of ordinary life and academic situations” (Richardson & Suinn 1972, p. 551). It is a multidimensional construct whose various types have been tested by other researchers, e.g., math learning anxiety, math testing anxiety (Abbreviated Math Anxiety Scale; AMAS; Hopko et al. 2003), math problem solving anxiety (Math Anxiety Questionnaire for Adults; MAQA; Szczygieł, 2021a). As various dimensions of math anxiety are usually positively and highly correlated to each other, math anxiety may also be treated as unidimensional (Single Item Math Anxiety Scale; SIMA; Núñez-Peña et al. 2014). Math anxiety begins in childhood and develops during the first years of primary school (Petronzi et al. 2019; Szczygieł & Pieronkiewicz 2022). It increases as the child gets older, peaking at 14 or 16 years old, followed by plateaus, but continuing through the school years and beyond (Yáñez-Marquina, & Villardón-Gallego 2017). Emotions accompanying learning mathematics are so intense that negative math attitude and high math anxiety may persist among adults many years after graduation (Hart & Ganley 2019; Szczygieł, 2021a, 2022).

Recent meta-analyses have clearly demonstrated a small-to-moderate negative association between math anxiety and math performance in children, adolescents, and adults (Barroso et al. 2021; Namkung et al. 2019; Zhang et al. 2019). The negative relationship between math anxiety and math performance is weaker in primary school children than in secondary school children and adults (Zhang et al. 2019). This relationship is observed regardless of the dimensions of math anxiety and the type of mathematical tasks.

## Numerical magnitude processing and math anxiety

Although numerical magnitude processing and math anxiety have often been tested as predictors of math performance, little attention has been paid to the relationship between them. Recently, Maloney et al. (2010, 2011) proposed the hypothesis that math anxiety is related to deficits in numerical magnitude processing. The development of math anxiety may be a result of a basic low-level deficit in numerical processing which in turn compromises the development of advanced mathematical skills. Maloney et al. (2010) indicated that math-anxious adults (AMAS) present higher reaction times in a visual enumeration task than their low math anxiety counterparts. In two follow-up studies, Maloney et al. (2011) demonstrated that high math anxiety adults (AMAS) have a stronger numerical distance effect on response time than low math anxiety individuals in a symbolic comparison task; this suggests that those with a high level of math anxiety have less precise numerical magnitude representations. Núñez-Peña & Suárez-Pellicioni (2014) tested high and low math anxiety adults (Math Anxiety Rating Scale, MARS, Richardson & Suinn 1972) using a single-digit comparison task. They revealed that numerical distance and size effects were marginally larger for the high math anxiety group of adults, thus supporting Maloney et al.’s hypothesis (2010). However, the hypothesis that numerical deficit underlies math anxiety (AMAS) was challenged in a study by Dietrich et al. (2015), who conducted a study with symbolic and non-symbolic comparison tasks (indicators: accuracy and reaction time for both, distance and size effects for both, Weber fraction for a non-symbolic task) in adults. Although they replicated previous findings showing that high math anxiety individuals had a larger distance effect in a symbolic comparison task than low math anxiety individuals, there was no relationship between math anxiety and non-symbolic comparison task indicators. Different results regarding symbolic processes were provided by Colomé (2019), who tested high and low math anxiety (MARS) groups of adults performing symbolic and non-symbolic comparison tasks and the counting Stroop task. The results indicated that high and low math anxiety groups did not differ in terms of accuracy, reaction time, Weber fraction, and numerical ratio effect in a non-symbolic task; they also did not differ in reaction time, numerical distance and size effects in symbolic tasks; and they also did not differ in accuracy, reaction time, and distance effect in the counting Stroop task. Núñez-Peña et al. (2019) are the only researchers who have checked whether math anxiety level (short MARS; Alexander & Martray 1989) is related to performance in the number line estimation task (two tasks – typical and easy: 0–100, 0–1,000; two tasks – untypical and difficult: 0–100,000, 267–367) in adults. They observed that math anxiety is negatively related to performance only in less familiar and more difficult number line estimation tasks, thus again challenging the hypothesis proposed by Maloney et al. (2010). Summing up, most results indicate that math anxiety is more related to symbolic magnitude processing, especially manipulation of large numbers, than to the processing of non-symbolic quantities. These results suggest that symbolic rather than non-symbolic representation is important for the formation of math anxiety.

## Numerical magnitude processing, math anxiety, and math performance

Studies by Maloney et al. (2010, 2011), Núñez-Peña & Suárez-Pellicioni (2014), Dietrich et al. (2015), Colomé (2019), and Núñez-Peña et al. (2019) focused on the relationship between magnitude processing and math anxiety, but not on math performance. However, other researchers have examined the relationship between all the aforementioned variables in adults, which to some extent enabled the verification of the hypothesis formulated by Maloney et al. (2010). Lindskog et al. (2017) tested adults using a non-symbolic intermixed comparison task, and the proportion of correct trials in the task was used as an ANS indicator. They revealed that math anxiety (revised MARS; Hopko 2003) mediates the relationship between ANS accuracy and math performance (arithmetic fluency test) as well as the relationship between math performance and ANS. Moreover, ANS accuracy predicted math anxiety and math anxiety predicted ANS, even though other variables were controlled for. Skagerlund et al. (2019) tested the relationship between ANS (a latent variable that consisted of one-digit and two-digit comparison tasks and reaction time as an indicator), math anxiety (Mathematics Anxiety Scale-UK; Hunt et al. 2011), and math performance (standardized mathematical test) in adults. They observed that symbolic magnitude processing mediates the relationship between math anxiety and math performance. Slightly different results were obtained by Maldonado Moscoso et al. (2020), who revealed that math anxiety (AMAS) mediates the link between ANS (measured by a non-symbolic comparison task with the Weber fraction as an indicator) and math performance (standardized mathematical test) in adults with high math anxiety. These researchers also found a significant correlation between ANS and math anxiety, but only in the high math anxiety group. However, their further study (Maldonado Moscoso et al. 2022) showed that the precision of numerosity estimation (Weber fraction) was negatively related to math anxiety (AMAS) and that math anxiety fully accounted for the relationship between ANS and math performance in adults. Inconsistent results were provided by Braham & Libertus (2018), who evaluated levels of non-symbolic magnitude processing (non-symbolic comparison task: accuracy as an indicator), math anxiety (MARS), and math performance (standardized mathematical test) in adults. They observed that ANS and math anxiety independently predict calculation, math fluency, and applied problem solving, but they interact only in the case of the last. Braham & Libertus (2018), concluded that better ANS may be a protective factor against the negative effect of math anxiety on math performance in certain types of math. In contrast, Silver et al. (2022) more recently showed that ANS (two non-symbolic comparison tasks, accuracy as an indicator) is not related to math anxiety (AMAS) in adults, and that math anxiety, but not ANS, predicts math performance in the structural equation model.

Previous studies also tested the relationship between numerical magnitude processing, math anxiety, and math performance in children. Cargnelutti et al. (2017) did not find any significant relationship between ANS (accuracy of non-symbolic comparison, addition, and estimation tasks) and math anxiety (Scale for Early Math Anxiety; Wu et al. 2012) in second grade children. In two studies on first- to third-grade children, Szczygieł (2021b) mostly found no relationship between non-symbolic magnitude processing (comparison task with accuracy as an indicator) and math anxiety (modified Abbreviated Math Anxiety Scale for Elementary Children; Szczygieł, 2019; Math Anxiety Questionnaire for Children; Szczygieł, 2020a). In a recent study, Sarı and Szczygieł (2023) observed that math anxiety (Math Anxiety Scale; Şentürk 2010) mostly does not mediate the relationship between mental number representation (error rate in number line estimation task) and math performance (standardized mathematical tests). However, they showed that a higher sum of errors in number line task performance is related to a higher level of math anxiety, and the accuracy of mental representation of numbers in high math anxiety children is a key factor contributing to math performance.

Findings regarding the relationship between symbolic and non-symbolic magnitude processing, math anxiety, and math performance are inconsistent. Because studies on children have largely failed to show a joint effect of numerical magnitude processing and math anxiety on math performance, and some studies in adults have found that such associations are observed in groups with high math anxiety, it can be assumed that the protective effect of numerical magnitude representations appears in individuals with a high level of math anxiety.

## Objectives and hypotheses of the present study

The main aim of our study was to test the hypothesis formulated by Maloney et al. (2010), namely that math anxiety is related to deficits in numerical magnitude processing and both variables interact to predict math performance. Because the results of previous studies on this topic have been inconsistent and used different methodology, we suppose that the relationship between numerical magnitude processing, math anxiety, and math performance may depend primarily on the different cognitive processes involved in processing numerical information (symbolic vs. non-symbolic; comparison vs. estimation) and type of their indicators (accuracy, reaction time, ratio effect, size effect, distance effect, and Weber fraction). Our assumption is based on the following premises. First, it has been observed that symbolic and non-symbolic systems are related (Piazza et al. 2004) or independent (Dietrich et al. 2015; Lyons et al. 2012, 2015); second, the processes of comparison and estimation have been found to be unrelated (Sasanguie & Reynvoet 2013); third, numerical magnitude indicators have been found to be weakly correlated or unrelated to each other (Krajcsi 2017; Krajcsi et al. 2022; Price et al. 2012; Smets et al. 2014). Therefore, the type of measurement and indicators used in a study may change the nature of the relationship between numerical magnitude processing, math anxiety, and math performance; they may also explain previously inconsistent results.

To test whether the relationship between numerical magnitude processing, math anxiety, and math performance depends on the cognitive resources involved in performing numerical tasks, we designed a study that included different types of measures of numerical processing (number line estimation task, symbolic and non-symbolic comparison tasks). In addition, we calculated numerous indicators for numerical comparison and estimation tasks (accuracy, reaction time, ratio effect, size effect, distance effect, and Weber fraction). We were interested in whether the results would be consistent regardless of the numerical tasks and indicators used. We expect that the strength of the relationships between numerical magnitude processing, math anxiety, and math performance will be greater in symbolic magnitude processing than in non-symbolic processing. Although previous studies have used various measures of math anxiety, we assume that this has not had a significant impact on the results because different dimensions of math anxiety are strongly related to each other (Oszwa 2020; Szczygieł, 2021a). However, we examined different types of math anxiety (math learning anxiety, math testing anxiety, math problem solving anxiety, general math anxiety), taking into account the multidimensional math anxiety index in the study (we created one math anxiety indicator; see Data Analysis section and Appendix, Tables 4 and 5). Our study focuses on adults because most of the previous conflicting research verified the hypothesis of Maloney et al. (2010) in this age group. We were also interested in studying the relationship between numerical magnitude processing, math anxiety, and math performance in adults because developmental and educational changes are less rapid at this stage of life compared to childhood. Finally, we expect that the combined effect of numerical magnitude processing and math anxiety on math performance will emerge primarily among high math anxiety individuals, of which there may be many among adults.

Based on the previous inconsistent research results, we formulated four hypotheses: weaker numerical magnitude processing is related to higher math anxiety (H1); stronger numerical magnitude processing is positively related to math performance (H2); higher math anxiety is related to lower math performance (H3); stronger numerical magnitude processing is positively related to math performance in those with a high level of math anxiety (H4).

## Method

### Participants

We recruited 121 people for the study but the results of two participants were excluded due to using the same pseudonym and overwriting the results. The final sample included 119 young adults (90 women, 29 men) between the ages of 18 and 32 (*M* = 21.42, SD = 2.99). Adults differed in their fields of education and profession. Participants declared that their high school class profile was related to science, technology, engineering, and mathematics (STEM*; N* = 52), humanities and social sciences (*N* = 42), and other fields (*N* = 25). Study or work in the field of STEM was declared by 40 people, humanities and social sciences by 60 people, and ‘other’ was indicated by 19 people. We used convenience sampling as participants were recruited through an advertisement posted on the nationwide internet platform olx.pl. The minimum number of subjects was determined with g*power a priori for the planned statistical analyses (*α* = 0.05, *β* = 0.80, *r* = 0.25, two-tails test). Because in some cases the result did not meet the criteria for a reliable indicator of the variable (see measurements), there are differences in the number of observations in the tasks.

### Measurements

#### Numerical magnitude processing

*Number Line Estimation Task* (NLE) is a computer task that measures the mental representation of numbers. Participants were presented with number lines bounded by 0 at the origin and 1000 at the endpoint, and Arabic digits were displayed above the line. The participants’ task is to mark the place corresponding to the given number (2, 5, 18, 34, 56, 78, 100, 122, 147, 150, 163, 179, 246, 366, 486, 606, 722, 725, 738, 754, 818, 938) on the line. We used the 22 numbers proposed by Opfer and Siegler (2007). After marking the location of a number, the next number is displayed, and so on (Schneider et al. 2018a). Participants were not provided with feedback during the research session. The stimuli were presented to subjects in random order. No time pressure was applied in the procedure. The indicator of NLE is the sum of errors (difference between target and marked point on the number line) in each trial. A higher error in NLE indicates a worse mental representation of numbers.

*The Non-Symbolic Comparison Task* (NS) is used to measure the accuracy of ANS. In each trial, two boards with white dots appear on the screen; the participants’ task is to choose the one with more elements. The choice is made by pressing the marked keys on the keyboard: "A" (for the left board) or "L" (for the right board). The boards contained 8, 10, 12, 13, 14, 18, 20, 22, 26, or 32 dots. The second board always contained 16 dots. The set size and set ratio were balanced, which means the same number of sets with a given number and the same number of sets for each ratio were displayed on the screen. The boards differed in the size of the dots. In the consistent condition, the larger set was marked with larger dots; in the inconsistent condition, the larger set had dots of smaller size. In both the training and the test sessions, the boards presented the consistent and inconsistent conditions equally and in random order, as recommended by Nuerk et al. (2004). There were 30 trials in both conditions, and each trial was repeated twice, giving a total of 120 trials in the test session. Each pair of boards was displayed for 7 s, followed immediately by the next pair. The task started with a training session (4 trials). The whole task was implemented using DMDX software (Forster & Forster 2003). MATLAB was used to generate the boards (Gebuis & Reynvoet 2011). Accuracy (NS ACC), reaction time (NS RT), numerical ratio effect calculated on RT (NS NRE), distance effect calculated on RT (NS NDE), size effect calculated on RT (NS NSE), and Weber fraction (*W*) were used as indicators of ANS. NS ACC was calculated as the sum of correct answers (9% errors). NS RT was based on correct answers, except for outliers (5% outliers by rule *M* ± 3*SD*; *M* = 1476.72 ms, SD = 959.04 ms). Slopes were calculated for NRE and NDE. NSE was calculated as mean RT differences between large and small numbers in the constant distance between numbers (distance 2, 4, 6). Hypotheses were tested using data from participants in which the effect was demonstrated (see *N* in Table 1). Values used for slope calculation were as follows: NRE 0.5, 0.6, 0.7, 0.8, 0.9 (rounded to the decimal by mathematical rules), NDE 2, 3, 4, 6, 8, 10, 16. To make sure that numerical effects were observed, we conducted a one-sample *t*-Student test that compared the mean effects to 0. In each case, the difference was significant (*p* < 0.001), thus showing NS NRE (${t}_{\left(116\right)}=$ 16.87), NS NDE (${t}_{\left(118\right)}=$ − 17.26), and NS NSE (${t}_{\left(74\right)}=$ 9.08) effects. The Appendix includes figures (Figs. 4 and 5) for NS NRE and NS NDE effects (there is no NSE as it was calculated for differences between two conditions, no slopes). *W* for individuals was calculated using Pica et al. (2004) rules. More precise ANS is reflected by more points in the comparison task (NS ACC), shorter reaction time (NS RT), weaker numerical ratio effect slope (NS NRE), weaker distance effect slope (NS NDE), smaller differences in RT between large and small numbers (NS NSE), and higher Weber fraction (*W*). The numerical representation is more precise as the NRE and NDE slope approaches zero. In the case of NRE, a higher positive slope means worse accuracy of numerical representation. In the case of NDE, a higher negative slope reflects worse accuracy of numerical representation. We calculated split-half reliability for following indicators: NS ACC *r* = 0.67, *p* < 0.001, *N* = 119, NS RT *r* = 0.97, *p* < 0.001, *N* = 119, NS NRE *r* = 0.57, *p* < 0.001, *N* = 109, NS NDE *r* = 0.52, *p* < 0.001, *N* = 112, NS NSE *r* = 0.42, *p* < 0.01, *N* = 38.

*The Symbolic Comparison Task* (S) is similar to NS with the exception that numbers were used instead of dots. Two-digit numbers were used in the range 21 to 98. 104 research trials were used, and two training trials preceded the research session. The procedure of the test was analogous to that described above. Accuracy (S ACC), reaction time (S RT), numerical ratio effect calculated on RT (S NRE), distance effect calculated on RT (S NDE), and numerical size effect calculated on RT (S NSE) were used as indicators of symbolic magnitude representation. We calculated S ACC as the sum of correct answers (2% errors). NS RT was calculated on correct answers except outliers (2% outliers in accordance with rule *M* ± 3SD; *M* = 892.27 ms, SD = 299.79 ms). The hypotheses were tested based on data from participants in which the effect was demonstrated (see *N* in Table 1). Values used for slope calculation for NRE were 0.3, 0.4, 0.6, 0.7, 0.8, 0.9, 1 (rounded to the decimal in accordance with mathematical rules). For NDE, the values used were 10, 20, 30, 40, 50, 60, 70 (rounded to the decimal in accordance with mathematical rules). The indicator for NSE was calculated as the difference in RT between the higher number and the lower number in number pairs with a fixed-unit distance. Again, we conducted a one-sample *t*-Student test to compare the mean effects to 0. In each case, the difference was significant (*p* < 0.001), thus showing S NRE (${t}_{\left(117\right)}=$ 27.5), S NDE (${t}_{\left(117\right)}=$ − 26.51), and S NSE (${t}_{(89)}=$ 13.71) effects. Figures for S NRE and S NDE effects (there was no S NSE as it was calculated for differences between two conditions, not slopes) are presented in the Appendix (Figs. 6 and 7). More precise ANS is reflected by more points in the comparison task (S ACC), shorter reaction time (S RT), weaker numerical ratio effect slope (S NRE), weaker distance effect slope (S NDE), and smaller differences between large and small numbers in RT (S NSE). As in NS, the numerical representation is more precise as the NRE and NDE slope approaches zero. In the case of NRE, a higher positive slope means worse accuracy of numerical representation; in the case of NDE, a higher negative slope reflects worse accuracy of numerical representation. We established split-half reliability for all indicators: S ACC *r* = 0.51, *p* < 0.001, *N* = 119, S RT *r* = 0.97, *p* < 0.001, *N* = 119, S NRE *r* = 0.30, *p* < 0.001, *N* = 113, S NDE *r* = 0.14, *p* = 0.198, *N* = 92, S NSE *r* = 0.19, *p* = 0.264, *N* = 37.

#### Math anxiety

We wanted to consider the multidimensionality of math anxiety in the study, so we used several research tools and then created one math anxiety indicator (see Data Analysis and Appendix).

*The Single-Item Math Anxiety Scale* (SIMA) measures general math anxiety (Núñez-Peña et al. 2014). The scale consists of one question: "On a scale of 1 to 10, how mathematically anxious are you?". Respondents answer on a 10-point scale, where 1 means "no anxiety" and 10 means "very anxious". SIMA has good psychometric properties and is considered an interesting alternative to longer questionnaires measuring math anxiety. SIMA test–retest reliability was found to be *r* = 0.72 in a Polish sample of adults (Szczygieł, 2022). As the scale contains 1 item, reliability was not estimated in the current research.

*Math Anxiety Questionnaire for Adults* (MAQA; Szczygieł, 2021a) is a non-school-dependent questionnaire, which means it has no items related to formal mathematics education. Its purpose is to measure the level of anxiety associated with solving mathematical problems in everyday and academic life. MAQA was designed to measure math anxiety in adults in an ecologically valid way. The questionnaire requires referring to various situations related to mathematics by marking a response on a 4-point scale, where 1 means "I definitely do not feel anxiety" and 4 means "I definitely feel anxiety". The questionnaire includes 19 items. A higher sum of points in MAQA means a higher level of math anxiety. The MAQA has satisfactory psychometric properties (Szczygieł, 2021a). McDonald’s ω calculated for latent MAQA factor was 0.87 in the current study.

*The Abbreviated Math Anxiety Scale* (AMAS) is a 9-item questionnaire on math anxiety, designed by Hopko et al. (2003) and adapted to the Polish context by Cipora et al. (2015). The AMAS total score includes two components: anxiety related to learning mathematics (AMAS-L) and anxiety related to being tested in mathematics (AMAS-T). Responses are given on a 5-point Likert scale, where 1 means low anxiety and 5 means high anxiety. A higher sum of points on each subscale indicates higher learning and testing math anxiety, respectively. The questionnaire is characterized by satisfactory psychometric properties in adults. The latent factor reliability for the Learning scale was ω = 0.74, and for the Testing scale it was ω = 0.82.

#### Math performance

*Math Performance Test* (MATH) was used to test math competencies in adults. There are no standardized math tests for adults in Poland, so participants’ math performance was measured using self-prepared tasks. The tasks were selected and compiled based on knowledge and competencies that should be mastered by high school students (counting, geometry). Each math task was a word problem formulated in an everyday life context. This decision was made because adults are diverse in terms of age and experience in learning mathematics. The test consists of 20 multiple choice close-ended questions. 3% of responses were removed as outliers (results from four participants). A higher total score indicates better math performance. The average level of test difficulty for the whole group was 0.83, which means that test was rather easy. The reliability of the test was Cronbach’s *α* = 0.78.

## Procedure

The study took place in the laboratory and participants were tested individually by two researchers who were familiar with the procedure. The study was approved by the ethics committee. Participants were informed about the purpose of the study and the possibility of asking questions and withdrawing from participation; they were briefed on the GDPR rules and filled out a consent form. Then, the actual procedure started, with the following tasks being carried out in sequence: NS, NLE, S. Each task was preceded by instructions and a short training session. Next, the subject filled out the SIMA, AMAS, and MAQA questionnaires. The final part of the study was MATH, after which the subject filled out self-reported personal information (gender, education, profession). Finally, participants performed verbal and visuospatial working memory tasks, whose results are not presented in this study. Participants received remuneration in the form of a voucher to use at the selected store. The reward amount (€8–12) was random and was not dependent on the level of task performance.

## Results

### Data analysis

The descriptive statistics, zero-order correlation, and moderation analyses were prepared in IBM SPSS Statistics 29 and PROCESS macro (Model 1; Hayes 2017). Before hypothesis testing, we created one indicator of math anxiety (MA) from the sum of points in all math anxiety questionnaires (SIMA, MAQA, AMAS). Before that, we tested whether math anxiety can be treated as one factor using CFA (R, *Lavaan* package, Rosseel 2012) with a maximum likelihood estimator. To evaluate the model’s fit, we used the following interpretation criterion: ${\chi }^{2}$ should be non-significant, RMSEA and SRMR should be < 0.08, and CFI and TLI should be > 0.95 (Hu & Bentler, 1999; Kline 2016).

We tested the model in which MA was a superior latent factor over four factors: SIMA (observed variable—only one item), MAQA (latent variable consisted of 19 items, λ from 0.31 to 0.67, *p* < 0.001), AMAS Learning (latent factor consisted of 5 items, λ from 0.48 to 0.71, *p* < 0.001), and AMAS Testing (latent factor consisted of 4 items, λ from 0.67 to 0.80, *p* < 0.001). Factor loadings for each item in all scales are provided in Table 4 in the Appendix. Correlations between all math anxiety scales are presented in Table 5 in the Appendix. The results confirmed the unidimensional model: ${\chi }_{(374)}^{2}$= 289.41, *p* = 1.00, CFI = 1.00, TLI = 1.03, RMSEA = 0 [90% CI 0, 0], SRMR = 0.08. All paths were well fitted to the math anxiety factor: SIMA λ = 0.86, *p* < 0.001, MAQA λ = 0.75, *p* < 0.001, AMAS Learning λ = 0.80, *p* < 0.001, AMAS Testing λ = 0.78. The reliability of the latent factor for MA was ω = 0.85.

## Descriptive statistics and correlation analyses

Descriptive statistics of all examined variables are presented in Table 1.

**Table 1:** Descriptive Statistics

	*N*	*M*	SD	Sk	*K*	Min	Max
NS ACC	119	106.41	6.94	− 1.72	5.20	74.00	120.00
NS RT	119	1374.11	426.04	.62	.03	631.05	2616.66
NS NRE	117	1318.93	845.52	.63	.04	9.99	4045.38
NS NDE	119	− 38.75	24.50	− .74	.78	− 130.01	− .04
NS NSE	75	145.91	139.10	1.56	2.03	1.21	606.63
NS W	119	.13	.06	.14	.84	0	.31
S ACC	119	98.78	3.25	− 3.35	19.82	76.00	102.00
S RT	119	867.18	129.98	.85	1.90	659.42	1444.70
S NRE	118	242.41	95.76	.40	.24	35.67	544.14
S NDE	118	− 2.46	1.01	.09	− .46	− 4.75	− .10
S NSE	90	127.24	88.07	1.81	6.32	4.53	567.07
NLE	119	706.14	277.97	.77	.24	242.00	1506.00
MA	119	60.68	15.49	.21	− .58	32.00	97.00
MATH	115	17.14	2.20	− .94	.72	10.00	20.00

NS: non-symbolic comparison task, S: symbolic comparison task, ACC: accuracy, RT: response time, NRE: numerical ratio effect, NDE: numerical distance effect, NSE: numerical size effect, *W*: Weber fraction, NLE: number line estimation task, MA: math anxiety, MATH – math performance

We tested whether MA is positively related to NS/S RT, NS/S NRE, NS/S NSE, NLE error; MA is negatively related to NS/S ACC, NS/S NDE, NS *W* (H1); MATH is negatively related to NS/S RT, NS/S NRE, NS/S NSE, NLE error, MATH is positively related to NS/S ACC, NS/S NDE, NS *W* (H2); MA and MATH are negatively related (H3); a higher level of MATH will be related to higher NS/S ACC, shorter NS/S RT, weaker NS/S NRE, weaker NS/S NDE, weaker NS/S NSE, stronger Weber fraction (*W*), and lower NLE error in those with a high level of MA (H4). The results are presented in Table 2.

**Table 2:** Correlation Matrix

		1	2	3	4	5	6	7	8	9	10	11	12	13
1	NS ACC													
2	NS RT	− .37***												
3	NS NRE	− .11	.68***											
4	NS NDE	.12	− .66***	− .93***										
5	NS NSE	-.12	.40***	.57***	− .45***									
6	NS W	− .57***	− .07	− .19*	.16	.01								
7	S ACC	− .04	.07	.14	− .10	.23	− .07							
8	S RT	− .03	.27**	.11	− .09	− .02	− .08	− .34***						
9	S NRE	− .02	− .08	− .06	.04	− .11	− .04	− .14	.07					
10	S NDE	.12	.01	.04	-.02	− .07	.03	− .05	.01	− .70***				
11	S NSE	− .01	.15	.01	.02	− .08	.08	− .39***	.31**	.15	− .23*			
12	NLE	− .17	− .18*	− .26**	.21*	− .15	.12	− .01	.07	.10	− .15	.03		
13	MA	.01	− .19*	− .28**	.22*	− .25*	.05	− .26**	.24**	.21*	− .18*	.20	.33***	
14	MATH	.04	.09	.14	− .09	.06	− .08	.12	− .03	− .16	.10	− .11	− .31***	− .44***

NS: non-symbolic comparison task, S: symbolic comparison task, ACC: accuracy, RT: response time, NRE: numerical ratio effect, NDE: numerical distance effect, NSE: numerical size effect, *W*: Weber fraction, NLE: number line estimation task, MA: math anxiety, MATH: math performance

^***^p < .001, **p < .01, *p < .05, Pairwise deletion was applied (*N* = 58–119).

We observed that MA is negatively and weakly related to NS RT (opposite to H1), NS NRE (opposite to H1), and NS NSE (opposite to H1). MA is positively and weakly related to NS NDE (opposite to H1). No relationship was observed between MA and NS ACC (H1 not confirmed, *p* = 0.91), and MA and NS *W* (H1 not confirmed, *p* = 0.59)*.* Then, we observed a weak negative relationship between MA and S ACC (H1 confirmed) and between MA and S NDE (H1 confirmed). We observed a weak positive relationship between MA and S RT (H1 confirmed) and between MA and S NRE (H1 confirmed). Between MA and S NSE we did not observe a significant relationship (H1 not confirmed, *p* = 0.06). A weak positive relationship was observed between MA and NLE error (H1 confirmed).

H2 was confirmed only in the case of the relationship between MATH and NLE error (negative and moderate relationship). No significant correlations were observed between the MATH and NS indicators and between the MATH and S indicators.

MA and MATH were found to be negatively and weakly related, which confirms H3.

## Interaction analysis

**Fig. 1:**
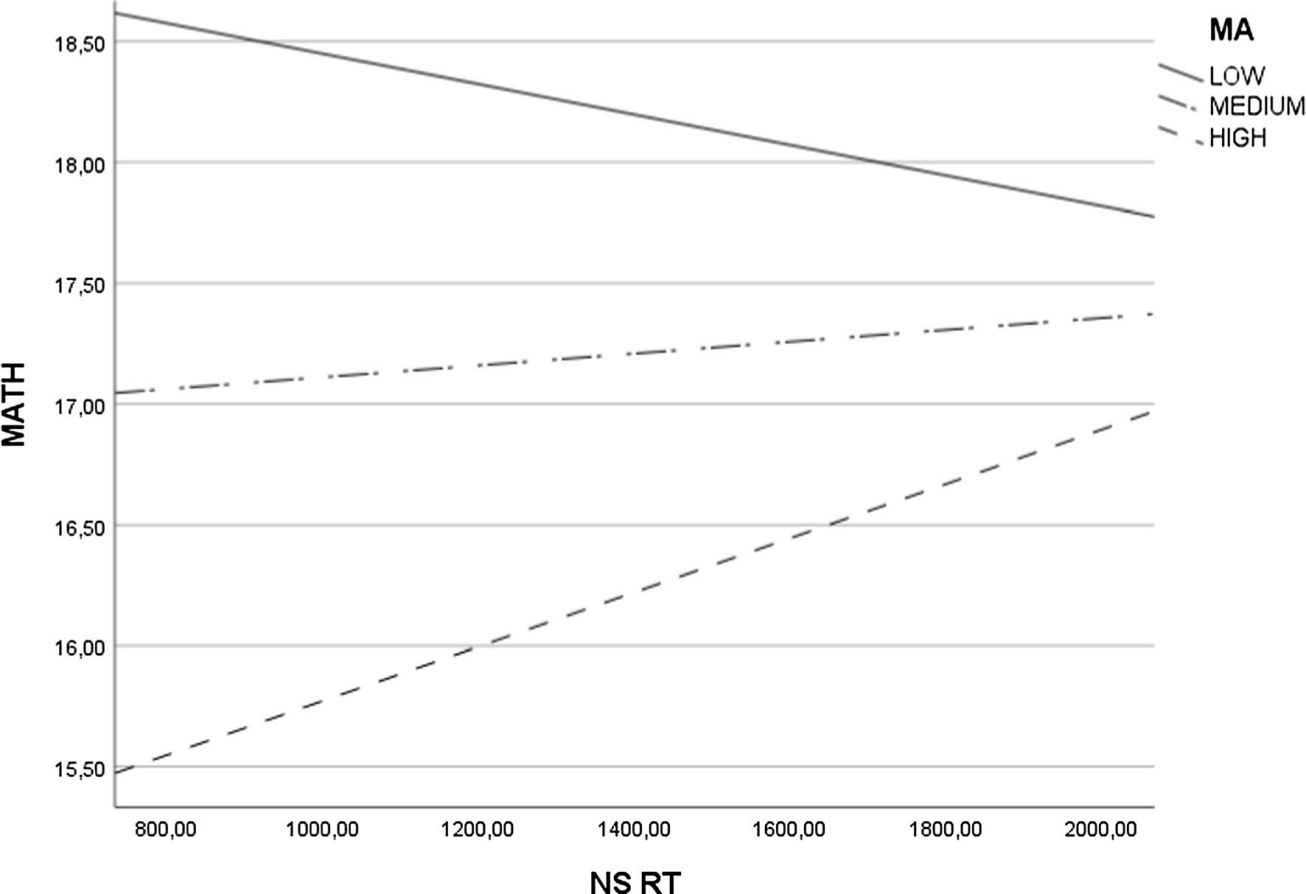
Moderation Effect of Math Anxiety (MA) on the Relationship between Reaction Time in Non-Symbolic Comparison Task (NS RT) and Math Performance (MATH). Positive Relationship between NS RT and MATH is Observed in Adults with High level of MA

**Fig. 2:**
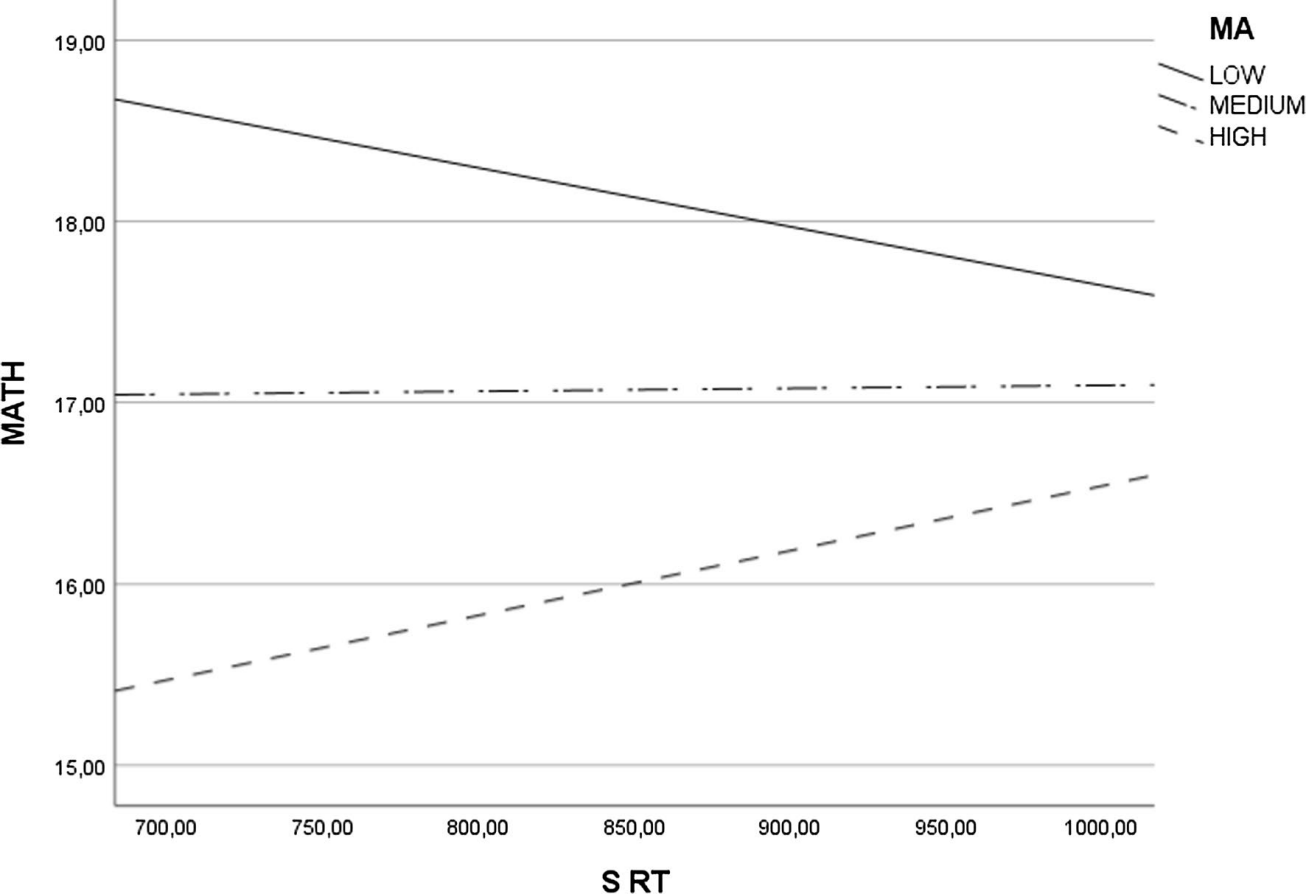
Moderation Effect of Math Anxiety (MA) on the Relationship between Reaction Time in the Symbolic Comparison Task (S RT) and Math Performance (MATH). A Positive Relationship between S RT and MATH is Observed in Adults with High level of MA

**Fig. 3:**
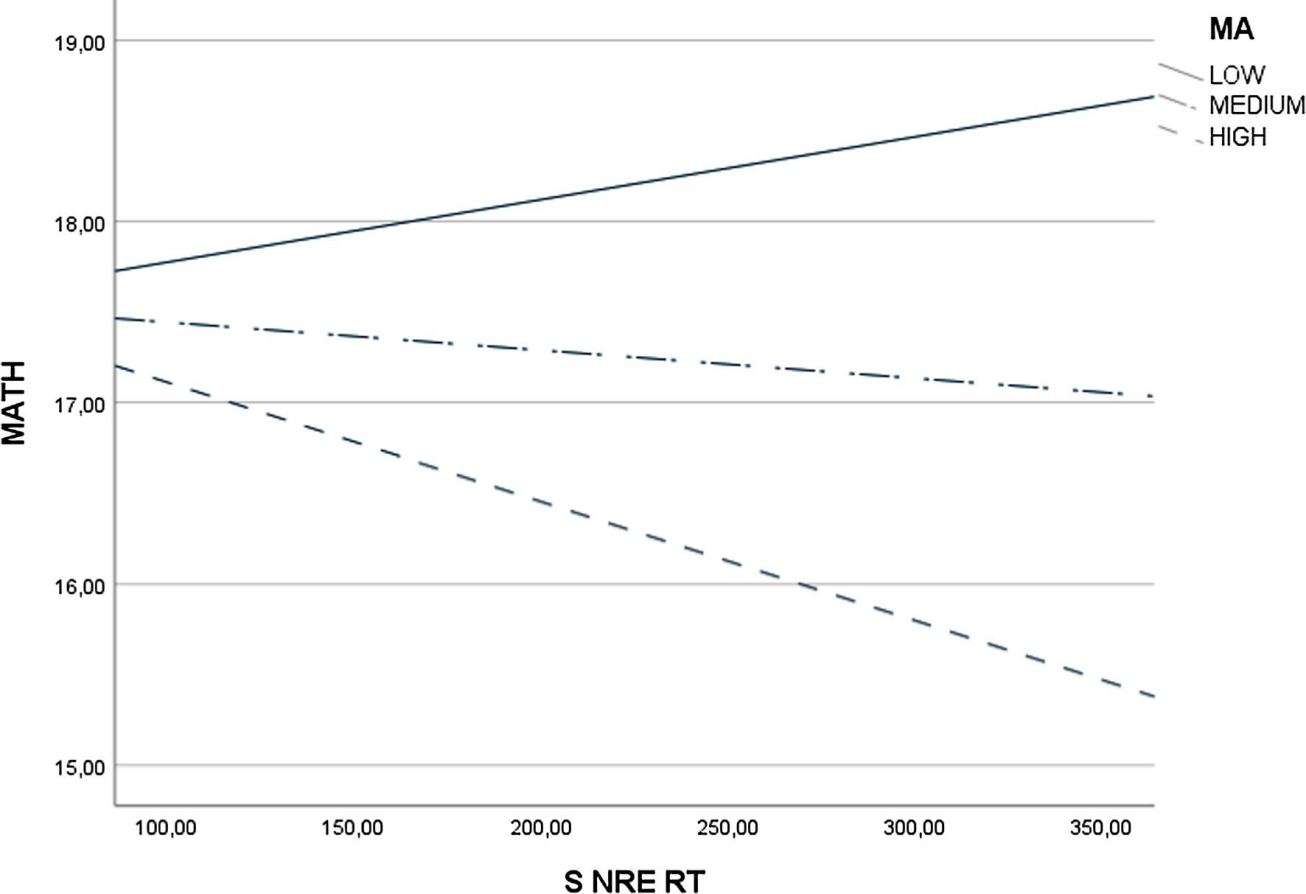
Moderation Effect of Math Anxiety (MA) on the Relationship between Numerical Distance Effect on Reaction Time in the Symbolic Comparison Task (S NRE RT) and Math Performance (MATH). A Positive Relationship between S NRE RT and MATH is Observed in Adults with High level of MA

**Fig. 4:**
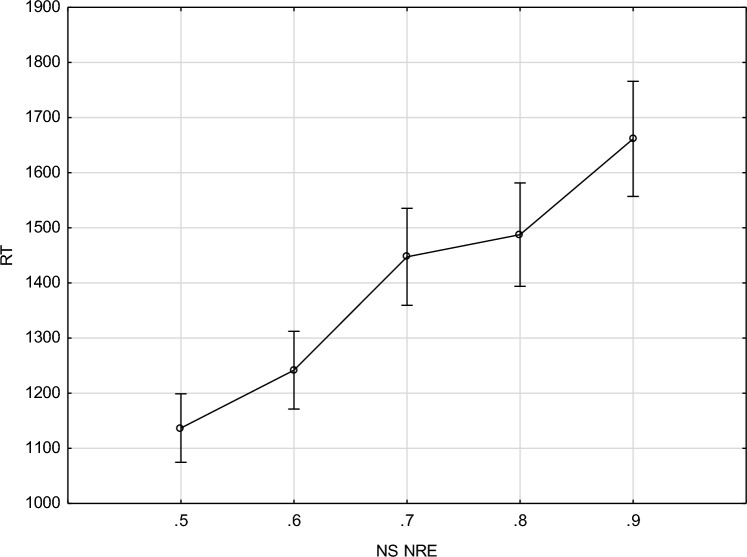
Numerical Ratio Effect (NRE) on Reaction Time (RT) in Non-Symbolic Comparison Task (NS)

**Fig. 5:**
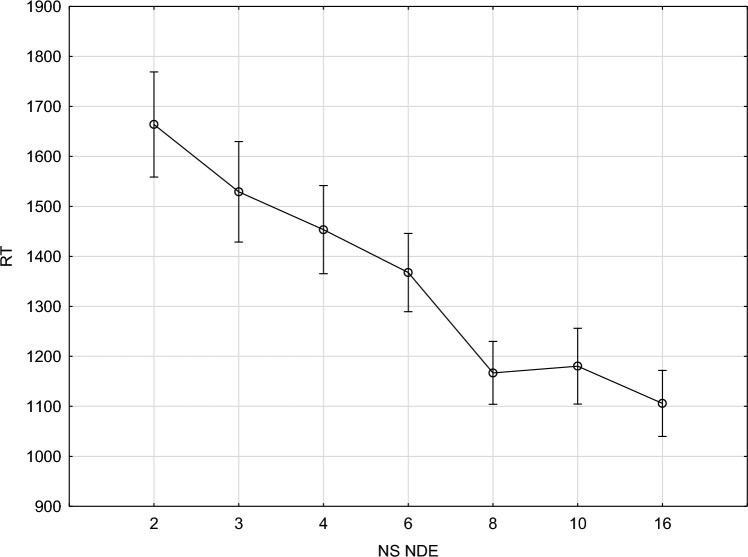
Numerical Distance Effect (NDE) on Reaction Time (RT) in Non-Symbolic Comparison Task (NS)

**Fig. 6:**
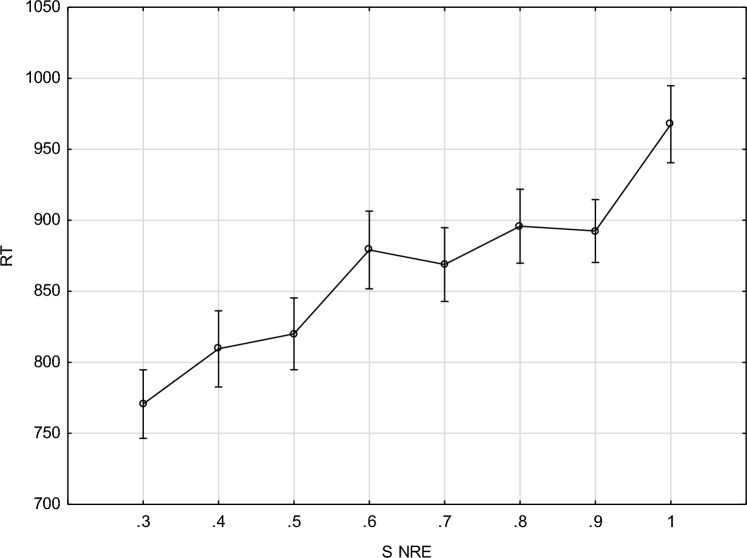
Numerical Ratio Effect (NRE) on Reaction Time (RT) in Symbolic Comparison Task (S)

**Fig. 7:**
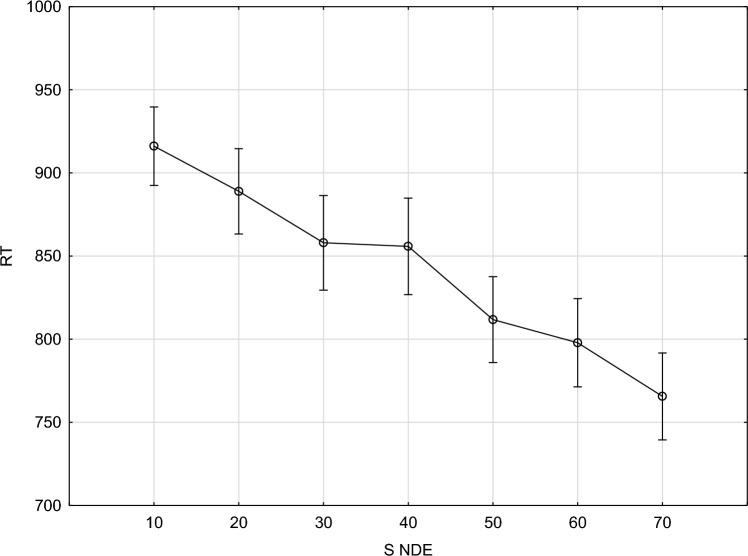
Numerical Distance Effect (NDE) on Reaction Time (RT) in Symbolic Comparison Task (S)

In the second step of analysis, we tested whether MA moderates the relationship between various indicators of numerical magnitude processing and MATH (see Table 3, Figs. 1, 2 and 3).

**Table 3:** Moderation of Math Anxiety between Numerical Magnitude Processing and Math Performance

	NS ACC x MA		S ACC x MA
ANOVA	*F*(3,111) = 9.64, *p* < .001, *N* = 115, ${R}^{2}$ = .21	ANOVA	*F*(3,111) = 9.33, *p* < .001, *N* = 115, ${R}^{2}$ = .20
Main effect NS ACC	*b* = .11, *se* = .10, *p* = .25 [− .08, .31]	Main effect S ACC	*b* = − .23, *se* = .31, *p* = .46 [− .85, .39]
Main effect MA	*b* = .12, *se* = .17, *p* = .49 [− .22, .46]	Main effect MA	*b* = − .42, *se* = .45, *p* = .36 [− 1.30, .47]
Interaction effect	*b* = .00, *se* = .00, *p* = .29 [− .01, .00]	Interaction effect	*b* = .00, *se* = .00, *p* = .43 [− .01, .01]
J-N significance region	–	J-N significance region	–

	NS RT x MA		S RT x MA
ANOVA	*F*(3,111) = 11.05, *p* < .001, *N* = 115, ${R}^{2}$ = .23	ANOVA	*F*(3,111) = 12.98, *p* < .001, *N* = 115, ${R}^{2}$ = .26
Main effect NS RT	*b* = .00, *se* = .00, *p* = .04 [− .01, − .0001]	Main effect S RT	*b* = − .02, *se* = .01, *p* = .01 [− .03, .00]
Main effect MA	*b* = -.15, *se* = .04, *p* < .001 [− .22, − .07]	Main effect MA	*b* = − .30, *se* = .08, *p* < .001 [− .46, − .14]
Interaction effect	*b* = .00, *se* = .00, *p* = .03 [.00, .00]	Interaction effect	*b* = .00, *se* = .00, *p* < .001 [.00, .00]
J-N significance region	Above 88.60 points in MA (HMA, Fig. 1)	J-N significance region	Above 75.35 points in MA (HMA, Fig. 2)

	NS NRE x MA		S RT x MA
ANOVA	*F*(3, 110) = 10.13, *p* < .001, *N* = 114, ${R}^{2}$ = .22	ANOVA	*F*(3,110) = 11.97, *p* < .001, *N* = 114, ${R}^{2}$ = .25
Main effect NS NRE	*b* = .00, *se* = .00, *p* = .08 [.00, .00]	Main effect S NRE	*b* = .02, *se* = .01, *p* = .03 [.00, .03]
Main effect MA	*b* = -.10, *se* = .02, *p* < .001 [− .14, -.05]	Main effect MA	*b* = .01, *se* = .03, *p* = .72 [− .05, .07]
Interaction effect	*b* = .00, *se* = .00, *p* = .06 [.00, .00]	Interaction effect	*b* = .00, *se* = .00, *p* = .01 [.00, .00]
J-N significance region	–	J-N significance region	Above 68.84 points in MA (HMA, Fig. 3)

	NS NDE x MA		S NDE x MA
ANOVA	*F*(3,111) = 9.96, *p* < .001, *N* = 115, ${R}^{2}$ = .21	ANOVA	*F*(3,110) = 9.51, *p* < .001, *N* = 114, ${R}^{2}$ = .21
Main effect NS NDE	*b* = .04, *se* = .03, *p* = .16 [− .02, .10]	Main effect S NDE	*b* = − .53, *se* = .87, *p* = .55 [− 2.25, 1.20]
Main effect MA	*b* = − .09, *se* = .02, *p* < .001 [− .14, − .05]	Main effect MA	*b* = -.04, *se* = .03, *p* = .19 [− .11, .02]
Interaction effect	*b* = .00, *se* = .00, *p* = .15 [.00, .00]	Interaction effect	*b* = .01, *se* = .01, *p* = .48 [− .02, .04]
J-N significance region	–	J-N significance region	–

	NS NSE x MA		S NSE x MA
ANOVA	*F*(3,70) = 4.27, *p* < .001, *N* = 74, ${R}^{2}$ = .15	ANOVA	*F*(3,83) = 5.40, *p* = .002, *N* = 87, ${R}^{2}$ = .16
Main effect NS NSE	*b* = − .01, *se* = .01, *p* = .33 [− .02, .01]	Main effect S NSE	*b* = − .01, *se* = .02, *p* = .55 [− .04, .02]
Main effect MA	*b* = − .07, *se* = .02, *p* = .005 [− .12, − .02]	Main effect MA	*b* = − .07, *se* = .03, *p* = .02 [− .13, − .01]
Interaction effect	*b* = .00, *se* = .00, *p* = .35 [.00, .00]	Interaction effect	*b* = .00, *se* = .00, *p* = .60 [.00, .00]
J-N significance region	–	J-N significance region	–

	NS W x MA		NLE x MA
ANOVA	*F*(3, 111) = 9.37, *p* < .001, *N* = 115, ${R}^{2}$ = .20	ANOVA	*F*(3,111) = 11.37, *p* < .001,* N* = 115, ${R}^{2}$ = .24
Main effect NS W	*b* = − 10.46, *se* = 15.16, *p* = .49 [− 40.51, 19.59]	Main effect NLE	*b* = .00, *se* = .00, *p* = .21 [− .01, .00]
Main effect MA	*b* = -.08, *se* = .03, *p* = .02 [− .15, − .01]	Main effect MA	*b* = − .07, *se* = .03, *p* = .01 [− .13, − .02]
Interaction effect	*b* = .13, *se* = .24, *p* = .58 [-.34, .60]	Interaction effect	*b* = .00, *se* = .00, *p* = .49 [.00, .00]
J-N significance region	–	J-N significance region	–

NS: non-symbolic comparison task, S: symbolic comparison task, ACC: accuracy, RT: response time, NRE: numerical ratio effect, NDE: numerical distance effect, NSE: numerical size effect, *W*: Weber fraction, NLE: number line estimation task, MA: math anxiety, MATH: math performance, HMA: high math anxiety. Effects: unstandardized coefficients [with 95% CI], J-N: Johnson-Neyman significance region (the point at which the non-significant effect changes to significant effect).

The results mostly challenge hypothesis H4 by showing that more precise numerical magnitude processing is related to higher MATH in individuals with high MA in three out of twelve analyses. There are positive relationships between NS RT and MATH (see Fig. 1) and between S RT and MATH (see Fig. 2) in high MA participants. The results also showed that there is a negative relationship between S NRE RT and MATH (see Fig. 3) in high MA participants. The other tested interactions were non-significant (see Table 3).

## Discussion

Having high mathematical competencies is considered very important from an individual and socioeconomic point of view. Compared to those with lower levels, people with high mathematical competencies earn more, are more successful professionally (Estrada-Mejia et al. 2016, 2020), and are more likely to make better decisions regarding their education and health (Garcia-Retamero et al. 2019; Rivera-Batiz 1992; Reyna et al. 2009; Sobków et al. 2020). All over the world, great importance is attached to organizing an optimal educational system that enables the acquisition of strong mathematical competencies and helps those who have difficulties coping with math (Dyson et al. 2013; Ramirez et al. 2018). Previous results suggest that enhanced numerical magnitude processing (Honoré & Noël, 2016) and reduced math anxiety (Sammallahti et al. 2023) lead to better math performance. However, relatively little is known about the relationship between numerical magnitude processing and math anxiety.

Recently, Maloney et al. (2010, 2011) suggested that deficits in numerical magnitude processing are related to math anxiety, and both variables interact to predict math performance. Although numerical magnitude processing and math anxiety have been tested as individual predictors of math performance (Barroso et al. 2021; Cueli et al. 2019), the relationship between both variables and their joint effect on math performance has not been sufficiently explored, and existing studies have yielded conflicting results. The results of previous findings have revealed a significant (Sarı & Szczygieł, 2023) or non-significant (Cargnelutti et al. 2017; Szczygieł, 2021b) relationship between numerical magnitude processing and math anxiety in children, and a significant (Braham & Libertus 2018; Lindskog et al. 2017; Maloney et al. 2010, 2011; Maldonado Moscoso et al. 2020, 2022; Núñez-Peña & Suárez-Pellicioni 2014; Núñez-Peña et al. 2019; Skagerlund et al. 2019) or non-significant (Braham & Libertus 2018; Colomé, 2019; Dietrich et al. 2015; Maldonado Moscoso et al. 2020; Núñez-Peña et al., 2010; Silver et al. 2022) relationship in adults. Inconsistent results have also been observed with respect to the relationship between the joint effect of numerical magnitude processing and math anxiety on math performance, showing significant mediation/interaction in adults (Braham & Libertus 2018; Lindskog et al. 2017; Skagerlund et al. 2019; Maldonado Moscoso et al. 2020), non-significant effects in adults (Braham & Libertus 2018; Silver et al. 2022), and non-significant effects in children (Szczygieł, 2021b; Sarı & Szczygieł, 2023).

Given that researchers used different study designs, measurements, variable indicators, and methods of statistical analysis, we assumed that the observed differences in results might be due to methodological differences. Therefore, we examined whether the relationship between numerical magnitude processing, math anxiety, and math performance depends on different numerical tasks and their indicators. Essentially, we tested whether the relationships depend on different cognitive processes involved in solving symbolic vs. non-symbolic tasks, and estimation vs. comparison tasks. We also examined the extent to which numerical magnitude processing and math anxiety separately determine math performance in adults.

## Numerical magnitude processing and math anxiety relationship

We observed that when symbolic numerical tasks are used in analyses, the hypothesis formulated by Maloney et al. (2011) regarding the relationship between numerical magnitude processing and math anxiety was mostly confirmed. However, the findings from the non-symbolic task challenge the hypothesis that less precise magnitude representation is the basis for math anxiety development. Therefore, the results suggest that the relationship depends on the type of numerical magnitude measure and its indices (Mielicki et al. 2022), and thereby, on the cognitive processes engaged in processing symbolic and non-symbolic numerical representations.

We observed that greater error on the number line estimation task was associated with higher math anxiety, thus supporting the hypothesis that individuals with less precise representation of symbolic magnitude have higher level of math anxiety. Our results are consistent with previous findings in adults (Núñez-Peña et al. 2019) and children (Sarı & Szczygieł, 2023). To our knowledge, these two studies are the only ones that have examined the relationship between a symbolic number line estimation task and math anxiety. We believe that a less precise mental number line contributes to less math understanding and more math anxiety.

Moreover, almost all indicators of the symbolic comparison task were associated with greater math anxiety (lower accuracy, higher reaction time, larger numerical ratio and distance effects). The relationship between MA and larger numerical size effect was non-significant, but the power of the test for this effect was lower than the power of the test for the other numerical effects because fewer participants had a reliable numerical size effect in comparison to other numerical effects. Although we did not divide the sample into low and high math anxiety individuals, we observed a similar pattern of results to previous findings comparing such groups (Maloney et al. 2010 – reaction time; Maloney et al. 2011 – numerical distance effect; Núñez-Peña & Suárez-Pellicioni 2014 – numerical distance effects). We also obtained results consistent with Dietrich et al. (2015) regarding the relationship between the numerical distance effect and math anxiety. More accurate and faster processing of symbolic numerical quantities may be crucial for the effectiveness of performing mathematical tasks, and this effectiveness is associated with positive (success) or negative (failure) emotions. As we used two-digit numbers in the symbolic comparison task, we also should note that cognitive multi-step processes were engaged in comparison. Indeed, in accordance with previous findings (Verguts et al. 2005; Nuerk et al. 2015), likely one numerical system is used for exact small and approximate large numbers and second numerical system represents multidigit numbers. Therefore, the question arises whether our results support the hypothesis that basic or more advanced numerical processes are related to math anxiety.

Most of the indicators of the non-symbolic comparison task were related to math anxiety, but the direction of the correlation was opposite to the hypothesis. Lower reaction time, and lower numerical ratio and size effects were related to higher math anxiety. Numerical distance effect was positively related to higher math anxiety. No relationship was observed between math anxiety and the accuracy and Weber fraction. These results are mostly inconsistent with previous findings in adults (Dietrich et al. 2015 – accuracy, reaction time, numerical distance and size effect, Weber fraction; Colomé et al., 2019 – accuracy, reaction time, Weber fraction, and numerical ratio effect) which showed a non-significant relationship between the variables or a different direction of the relationship. However, our results are consistent with findings that accuracy in non-symbolic comparison task is not related to math anxiety in children (Cargnelutti et al. 2017; Szczygieł, 2021b). Because performance in the non-symbolic comparison task may be influenced by methodological factors (e.g., visual stimulus parameters, presentation duration time, set size; Dietrich et al. 2015), our surprising results are likely due to the low time pressure in the task. Thus, individuals with high math anxiety reacted faster than those with low math anxiety, which suggests that they wanted to reduce the time to decide. However, the results confirm Dietrich et al.’s (2015) hypothesis that the relationship between numerical magnitude tasks and math anxiety may depend on task-related decision-making processes (in this case, the trade-off between speed and accuracy resulting from the desire to complete the task as quickly as possible). We then observed that weaker ratio and size effects (both effects calculated from reaction time) were related to greater math anxiety. This means that better discrimination of similar patterns of dots, even at large magnitudes with the same distance effect, is related to higher math anxiety. These results suggest that math anxiety in adults may be driven primarily by factors other than basic numerical magnitude processing. Indeed, many factors have previously been identified as predictors of math anxiety (Szczygieł & Hohol 2024; Zhange et al., 2019). However, it should also be noted that the Weber fraction is considered the most adequate indicator of ANS (Krajcsi 2020; Pica et al. 2004; Price et al. 2012) and, in our study, *W* was not associated with math anxiety.

The fact that the relationship between numerical magnitude processing and math anxiety is task- and indicators-dependent is not surprising because previous findings (Bulthé et al. 2014; Honoré & Noël, 2016; Lyons et al. 2015) suggest that symbolic and non-symbolic quantities are encoded and processed differently. Previous research results suggest that non-symbolic numerical processing is directed by ANS, and such processes may be described interchangeably by numerical size and distance effects (both directed by ratio effect), while symbolic numerical processing is likely handled not by ANS but by an alternative representation (e.g., Verguts et al. 2005; Krajcsi 2017; Krajcsi et al. 2022). For example, in accordance with model of Krajcsi et al. (2022), the distance effect is directed by semantic distance of units, and size effect is led by symbols frequency. Our results support claims of distinct mechanisms directing processing of symbolic and non-symbolic magnitudes: all numerical effects (ratio, size, and distance effects) correlated significantly in non-symbolic format, ratio and distance, distance and size correlated to each other, but ratio and size effects did not correlate in the symbolic comparison task. Moreover, strength of correlations in non-symbolic magnitude processing was weaker than in the symbolic comparison task. Finally, a debate has emerged regarding the most accurate indicator that reflects the functioning of the ANS. Current perspectives lean towards considering Weber’s *W* as the most effective indicator for non-symbolic magnitude processing, especially when accounting for lapse rate and perceptual properties. However, it remains uncertain to what extent accuracy may be influenced by the lapse rate (Krajcsi et al. 2023). Therefore, the conclusion is that deficiencies in symbolic but not non-symbolic magnitude processing may be a risk factor for the development of math anxiety but further studies on the nature of numerical processing are needed.

## Numerical magnitude processing and math performance

We observed that more precise mental number representation is related to higher level of math performance, while more effective processing of symbolic and non-symbolic numerical magnitudes is not related to math performance. Greater estimation error in the number line task was moderately related to poorer mathematical performance, as is consistent with previous findings in children (Friso-van den Bos et al. 2015; Schneider et al. 2018a). Individuals with a more precise mental representation of numbers perform better on math tasks, likely due to the more accurate decision-making strategies used when judging proportions (Slusser & Barth 2017). It is worth mentioning that previous findings have also shown that math education improves the mental representation of numbers (Friso-van den Bos et al. 2015; Schneider et al. 2018a). It should therefore be noted that this relationship may be bidirectional. More accurate representation of numbers improves math performance, and better math understanding improves estimation skills (Friso-van den Bos et al. 2015). The fact that the sum of error in the number line estimation task was related to math performance while the non-symbolic and symbolic comparison task indicators were unrelated may be explained by the different cognitive processes involved in the estimation and comparison tasks (Li et al. 2018). The non-symbolic comparison task is viewed as a test of the basic ability to process numerical quantities. It is likely that these skills do not translate into math performance in adulthood due to the development of more complex math skills that affect math performance. Similarly, it can be assumed that adults have mastered digits below 100 in childhood, therefore the role of processing simple symbols in their math performance decreases (Li et al. 2018). A large error variance was observed in the estimation task and a low error rate was observed in the comparison tasks, which means that the NLE task differentiated individuals more than the NS and S comparison tasks. Previous research also suggests that estimation is more important than comparison in predicting math performance (Schneider et al. 2018b). Based on a simple estimation task, it is possible to identify students who have potential difficulties in understanding mathematics, therefore it would be strongly advisable to use this type of task in educational practice (Nosworthy et al. 2013). In summary, as is consistent with previous findings, we can conclude that the type of numerical representation measurement (comparison or estimation) may determine the significance and strength of the relationship between numerical magnitude processing and math performance (Li et al. 2018; Schneider et al. 2017; 2018a), probably because they involve different cognitive processes (Sasanguie & Reynvoet 2013).

## Math anxiety and math performance

In line with our expectations and previous findings (Barroso et al. 2021; Zhang et al. 2019), math anxiety and math performance were negatively related. High math anxiety in adults may have a negative effect on their academic outcomes (e.g., students may drop out of STEM education due to negative emotions and math-related failures; Beilock & Maloney 2015; Picha 2018) and on their environment (e.g., early childhood teachers and parents may, under certain circumstances, pass on math anxiety to children; Sarı & Hunt 2020; Szczygieł, 2020b). Although it is still debated whether poor math performance causes high math anxiety, high math anxiety determines poor math performance or whether such a relationship is reciprocal (Carey et al. 2016), the negative relationship between both variables is observed across all age groups, including adults. Therefore, interventions to reduce math anxiety and/or improve math performance in adults who need it (e.g., STEM students, parents, and early childhood teachers; Maloney, 2015; Casad et al. 2015) should be considered.

## The joint effect of numerical magnitude processing and math anxiety on math performance

The results largely challenge the hypothesis that numerical magnitude processing and math anxiety have a joint effect on math performance. However, we observed that longer reaction times in symbolic and non-symbolic comparison tasks and a smaller numerical ratio effect in the symbolic comparison task are related to better math performance in adults with high math anxiety. In our opinion, such results suggest that people with high math anxiety need more time to solve mathematical tasks correctly. Indeed, in adults with high math anxiety, as reaction times in non-symbolic and symbolic comparison tasks increase, accuracy on math tasks also increases. Moreover, better math performance depends on the ratio effect in the symbolic comparison task in adults with high math anxiety. Therefore, we hypothesize that people with high math anxiety need more time for solving math tasks, mainly when they operate on two-digit numbers that are close to each other.

Summarizing the results regarding the relationship between numerical magnitude processing, math anxiety, and math performance, we hypothesize that the key for better math performance is the accuracy of the mental number line. Additionally, people with high math anxiety need more time to process magnitudes and perform mathematical tasks. This hypothesis requires support in further research when time pressure is imposed on numerical magnitude processing and mathematical tasks. However, our results are consistent with those observed in children (Sarı & Szczygieł, 2023 – negative relationship between number line estimation task error and math performance in high math anxiety children; Szczygieł, 2021b – no mediation effect of non-symbolic and symbolic comparison task accuracy between math anxiety and math performance) and adults (Silver et al. 2022 – no interaction effect of accuracy of non-symbolic task and math anxiety on math performance; Skagerlund et al. 2019 – reaction time as indicator of symbolic comparison task mediated relationship between math anxiety and math performance). Results opposite to ours were observed in the study of Lindskog et al. (2017 – math anxiety mediated accuracy of non-symbolic comparison tasks and math performance) and Maldonado Moscoso et al. (2020 – math anxiety mediated the relationship between the Weber fraction and math performance in high math anxiety individuals; Maldonado Moscoso et al. 2022 – math anxiety fully accounted for the relationship between numerosity estimation precision and math abilities for all participants). However, numerical magnitude processing tasks in their study were presented under greater time pressure than in our study. Partially contradictory to our results were those obtained by Braham & Libertus (2018), who showed that accuracy in non-symbolic comparison tasks and math anxiety interact to predict math performance, but only for certain types of mathematical tasks. These results suggest that further studies should include various types of mathematical tasks.

## Limitations and further research directions

Although our study was carefully designed, it has some important limitations. First, we tested a relatively small sample of adults, mainly young women and students living in a big city. Because we recruited respondents through an advertisement, the group we examined was limited to people seeking paid psychology studies. Moreover, people with high math anxiety were likely to refrain from participating in a study involving solving math problems. It should also be noted that the selected hypotheses were tested with fewer participants because some participants did not reveal a reliable numerical effect (primarily a numerical size effect in the symbolic and non-symbolic comparison tasks) or they revealed a floor effect in math performance. However, the advantage of our study was that we examined a diverse group of adults in terms of educational experience (STEM, HS, other fields of studies and professions). Although our results confirm that differences between studies may be explained by methodological details (type of measure and indicators of numerical magnitude processing), further studies are needed to show what this looks like in children and adolescents. Indeed, it is well known that both non-symbolic and symbolic numerical representations and math anxiety develop over time (Friso-van den Bos et al. 2015; Petronzi et al. 2019). Therefore, the relationships between these constructs and between them and math performance may vary by age group. Moreover, the educational situation of children and adults is very different (e.g., children are obliged to attend math classes, while adults can, if they want, continue their studies in STEM), which also justifies conducting research in these groups. Foremost, the longitudinal study design with control for various confounding variables would be most appropriate to test the hypothesis of Maloney et al. (2010). However, in our study, as in previous studies, we examined the relationships between variables in a cross-sectional design. It should also be noted that verifying this hypothesis is not easy because developmental and educational processes also take place.

Second, we ensured that numerical magnitude processing and math anxiety were tested using a variety of measures, but we used only a single task to measure math performance. The difficulty level of this task, according to Dolna’s classification (2016), was quite easy. We conducted a pilot study to select tasks for the test and to check whether the tasks were understandable for adults, but we did not further test the properties of the tasks. An important challenge for future studies on psychological correlates of math performance among Polish adults is the development of a standardized multidimensional scale of mathematical performance. Nevertheless, it can be assumed that the role of numerical magnitude processing and math anxiety – as domain-specific correlates of math performance – should be revealed for most mathematical tasks (Barroso et al., 2021; Schneider et al. 2017; 2018a, 2018b; Zhang et al. 2019). The strength of the correlation between indices of numerical magnitude processing, math anxiety, and math performance depends not only on the true relationship, but also on the measurement error noise (Krajci, 2017). We observed that the reliability of the symbolic and non-symbolic comparison tasks varied between their indicators which may affect observed results. Although split-half reliability was satisfactory in most indicators and in line with previous studies (Price et al. 2012), we observed no relationship between results in two effects: S NSE and S NDE. This may be the result of a few trials contributing to a given unit, taking into account the numerous filters placed on the two-digit symbols to make these effects possible to count. Moreover, it is still discussed how to interpret numerical distance and size effects in multi-digit symbols as their comparison needs multi-step processing (Verguts et al. 2005; Nuerk et al. 2015). Because we used only one set of stimuli in the NLE task (the stimuli were not repeated and the task was performed only once), we were unable to determine the reliability of the task. Due to many computer-based tasks being used in numerical cognition research lacking documented reliability, it is unknown how the psychometric properties of the task have influenced many previous results. Therefore, providing such information is one of the greatest challenges for further research on numerical cognition.

Moreover, we did not control more-general cognitive skills (intelligence, execution functions) and anxiety (trait anxiety, test anxiety), which poses a challenge for further studies. We assume that controlling domain-general variables may weaken the tested relationships. Although numerical magnitude processing and math anxiety are math domain-specific variables, they are partially rooted in domain-general skills and emotions (Friso-van den Bos et al. 2014; Simms et al. 2016; Szczygieł, 2021a). As recently observed, gender, spatial anxiety, emotional stability, state anxiety, and test anxiety explain 61% of the variation in math anxiety in adults (Szczygieł & Hohol 2024), however, further research is required on the cognitive nature of numerical magnitude representations (Nelwan et al. 2021). Assuming that symbolic and non-symbolic processes of magnitude processing and comparison and estimation are independent (Dietrich et al. 2015; Guillaume et al. 2016; Lyons et al. 2012, 2015; Sasanguie & Reynvoet 2013), it is likely that they are determined to varying degrees by domain-general cognitive skills.

## Conclusions

The results mostly support the hypothesis proposed by Maloney et al. (2010) in this area, namely that defective processing of symbolic magnitude is related to math anxiety; however, the results question assumption in this area that non-symbolic processes underlie math anxiety. We observed that better numerical estimation (but not comparison processes) and lower math anxiety correlate with better math performance in adults but results regarding the joint effect of numerical magnitude processing and math anxiety on math performance were inconsistent. We observed a relationship between math performance and reaction time in a symbolic comparison task, between math performance and reaction time in non-symbolic comparison tasks, and between math performance and the numerical ratio effect in a symbolic comparison task only in high math anxiety individuals. The results therefore suggest that timing and ratio (in symbolic processing) are crucial to the relationship between numerical magnitude processing and math performance in individuals with high math anxiety. Therefore, our results, like those of Braham and Libertus (2018), suggest that in further studies on predictors of math performance, numerical magnitude processing and math anxiety should be considered together, and their joint effect on math performance should be further examined.

## Data Availability

Data are publicly available in the OSF repository: 10.17605/OSF.IO/ZA4WS

## Appendix

See Figs. 4, 5, 6 and 7.

See Tables 4 and 5.

**Table 4:** Descriptive Statistics and Factor Loadings

Items and Scales	*M*	*SD*	*Sk*	*K*	*λ*
MAQA	34.09	8.99	.58	.48	
MAQA1	1.86	.77	.48	− .51	.54***
MAQA2	1.42	.69	1.52	1.44	.42***
MAQA3	2.08	.95	.37	− .93	.43***
MAQA4	1.62	.83	1.17	.49	.52***
MAQA5	1.97	.89	.43	− .85	.67***
MAQA6	1.47	.66	1.27	1.17	.58***
MAQA7	1.92	.90	.64	− .48	.46***
MAQA8	1.77	.86	1.03	.51	.50***
MAQA9	1.71	.92	1.15	.32	.53***
MAQA10	1.91	1.03	.76	− .67	.61***
MAQA11	1.39	.74	2.04	3.78	.52***
MAQA12	1.66	.88	1.10	.20	.55***
MAQA13	2.68	1.02	-.26	− 1.02	.59***
MAQA14	2.18	1.05	.20	− 1.29	.54***
MAQA15	1.48	.76	1.65	2.40	.48***
MAQA16	1.52	.82	1.47	1.23	.45***
MAQA17	1.66	.86	1.04	.07	.41***
MAQA18	2.42	1.05	.13	− 1.15	.42***
MAQA19	1.34	.67	2.05	3.90	.31***
AMAS Learning	8.86	3.65	1.07	1.17	
AMAS1	1.40	.81	2.20	4.67	.50***
AMAS3	1.78	1.08	1.30	.79	.71***
AMAS6	2.02	1.12	.85	− .12	.59***
AMAS7	1.80	1.07	1.30	.86	.49***
AMAS9	1.86	1.09	1.08	.11	.70***
AMAS Testing	13.97	3.74	− .50	− .69	
AMAS2	3.13	1.20	− .13	− .79	.69***
AMAS4	3.88	1.07	− .89	.17	.67***
AMAS5	2.86	1.25	.04	− 1.11	.80***
AMAS8	4.10	1.11	− 1.02	− .10	.74***
SIMA	3.76	2.48	.69	− .59	

^***^*p* < .001, *N* = 119

**Table 5:** Zero-Order Correlations Between Math Anxiety Measurements

	SIMA	MAQA	AMAS learning
MAQA	.72***		
AMAS Learning	.73***	.69***	
AMAS Testing	.67***	.66***	.76***

^***^*p* < .001; MAQA, AMAS Learning, AMAS Testing – Latent Factor Scores, SIMA – Observed Score
